# Empirical Rescue Eradication Therapy for *Helicobacter pylori* Infection in Second and Subsequent Treatment Lines: Experience From 500 Cases of the Brazilian Registry on *H. pylori* Management (Hp‐BrazilReg)

**DOI:** 10.1111/hel.70077

**Published:** 2025-10-14

**Authors:** B. S. F. Sanches, O. P. Nyssen, S. R. Chaves, J. S. Veloso, L. S. Silva, J. R. Marinho, H. Okamoto, G. C. Couto, H. P. Breyer, C. S. Alencar, E. Comelli, L. S. Sousa, M. Horn, M. G. Massote, M. T. R. Loures, M. P. Vidal, R. V. Paula, L. T. Ribeiro, H. O. Galizzi, D. A. A. Terra, G. G. L. Cançado, B. P. Burmann, J. S. Caetano, L. F. Pena, M. A. Decanio, H. S. Souza, A. S. O. Kuniyoshi, L. R. Guedes, M. C. F. Passos, F. P. Marinho, I. Z. Bombassaro, A. G. Domingues, J. G. Barbosa, I. M. Nogueira, A. F. P. Ramos, D. R. Korman, T. B. Souza, M. C. Barbosa, D. Chinzon, L. L. Silva, A. Mantovani, A. H. A. Freitas, C. S. Poncinelli, M. A. Francato, J. N. Goncalves, P. Parra, A. Cano‐Català, L. Moreira, J. P. Gisbert, Luiz Gonzaga Vaz Coelho, the Hp‐BrazilReg

**Affiliations:** ^1^ Universidade Federal de Minas Gerais Belo Horizonte Brazil; ^2^ Biocor Instituto Belo Horizonte Brazil; ^3^ Gastroenterology Unit, Hospital Universitario de La Princesa Instituto de Investigación Sanitaria Princesa (IIS‐Princesa), Universidad Autónoma de Madrid (UAM), and Centro de Investigación Biomédica en Red de Enfermedades Hepáticas y Digestivas (CIBERehd) Madrid Spain; ^4^ Rede Mater Dei de Saude Belo Horizonte Brazil; ^5^ Hospital Santa Helena Brasilia Brazil; ^6^ Hospital Adventista de Manaus Manaus Brazil; ^7^ Universidade Estadual de Ciências da Saúde de Alagoas Maceio Brazil; ^8^ Centro Medico Oxford São Bento do Sul Brazil; ^9^ Centro de Consultas Especializadas Contagem Brazil; ^10^ Hospital de Clinicas/Universidade Federal do Rio Grande do Sul Porto Alegre Brazil; ^11^ Clinicas Reunidas São Victor Rio de Janeiro Brazil; ^12^ Clinica Castromed Castro Brazil; ^13^ Coopclin Recife Brazil; ^14^ Clinica Endocentro Florianopolis Brazil; ^15^ Unidade de Saúde Santos Brazil; ^16^ Hospital Nossa Senhora das Graças Curitiba Brazil; ^17^ Hospital Ana Costa Santos Brazil; ^18^ Private Practice Aracaju Brazil; ^19^ Hospital Universitário Prof. Alberto Antunes Maceio Brazil; ^20^ Hepscan Belo Horizonte Brazil; ^21^ Instituto Alfa de Gastroenterologia/Hospital das Clínicas/Ebserh‐UFMG Belo Horizonte Brazil; ^22^ Hospital Militar de Minas Gerais Belo Horizonte Brazil; ^23^ MedPlus Porto Alegre Brazil; ^24^ Hospital São Rafael Salvador Brazil; ^25^ Socor Belo Horizonte Brazil; ^26^ Hospital Português Salvador Brazil; ^27^ Universidade Federal Do Rio de Janeiro Belo Horizonte Brazil; ^28^ Universidade Estadual de Maringá Maringá Brazil; ^29^ Santa Casa de Misericordia de Porto Alegre Porto Alegre Brazil; ^30^ Private Practice Rio de Janeiro Brazil; ^31^ Santa Casa de Barretos Barretos Brazil; ^32^ Private Practice São Paulo Brazil; ^33^ Grupo Santa Casa de Belo Horizonte Belo Horizonte Brazil; ^34^ Faculdades Integradas de Patos Patos Brazil; ^35^ Private Practice Arapiraca Brazil; ^36^ Universidade de São Paulo São Paulo Brazil; ^37^ Monte Sinai Hospital e Maternidade Juiz de Fora Brazil; ^38^ Universidade Do Vale Do Rio dos Sinos Porto Alegre Brazil; ^39^ Unimed Sul Capixaba Cachoeiro Do Itapemirim Brazil; ^40^ Biogastro Belo Horizonte Brazil; ^41^ Hospital Municipal Antonio Giglio Osasco Brazil; ^42^ Gastrointestinal Oncology, Endoscopy and Surgery (GOES) Research Group, Althaia Xarxa Assistencial Universitària de Manresa Institut de Recerca i Innovació en Ciències de la Vida i de la Salut de la Catalunya Central (IRIS‐CC) Manresa Spain; ^43^ Department of Gastroenterology, Hospital Clínic de Barcelona, Centro de Investigación Biomédica en Red en Enfermedades Hepáticas y Digestivas (CIBERehd), Institut D'investigacions Biomèdiques August Pi i Sunyer (IDIBAPS) University of Barcelona Barcelona Spain

**Keywords:** empirical treatment, *Helicobacter pylori*, registry, retreatment, treatment failure

## Abstract

**Background:**

The effectiveness of anti*‐H. pylori* treatment diminishes with therapy failure, making regional performance understanding crucial.

**Objective:**

To evaluate the effectiveness of empirical therapy in second‐line and subsequent treatments in Brazil.

**Methods:**

A multicenter, prospective, noninterventional registry assessed *H. pylori* management outcomes by Brazilian gastroenterologists (Hp‐BrazilRe, Hp‐WorldReg's partner). Data were registered at e‐CRF AEG‐ReCap from March 2022 to October 2024 and analyzed via modified intention‐to‐treat (mITT) methodology. Data were subject to quality review.

**Results:**

572 patients (mean age 52 years, 64% women) were included. The primary treatment indications were dyspepsia (64%) and gastroduodenal ulcer (9.2%). Among them, 67% underwent second‐line therapy, while 33% received third‐line or subsequent treatments. Proton‐pump inhibitors (PPIs) were administered at low (40%), standard (10%), and high doses (24%), with vonoprazan used in 26% of cases. The overall eradication rate for second‐line treatment was 74%, with the most common regimen being triple therapy (PPI + amoxicillin + levofloxacin), achieving 73% eradication for 14 days and 57% for 10 days. Adding bismuth to the 14‐day regimen increased effectiveness to 100% (*p* = 0.016). In third‐line therapy, a regimen of PPI‐bismuth‐tetracycline‐metronidazole yielded an 87% cure rate. The fourth‐line dual therapy with amoxicillin‐vonoprazan achieved 100% eradication, while bismuth‐quadruple therapy showed similar results. Dual therapy with vonoprazan and amoxicillin was also effective in fifth‐line treatments, achieving 100% effectiveness. Mild adverse events occurred in 23% of patients, with nausea being the most common (14%), and compliance was 99%.

**Conclusion:**

In Brazil, the overall effectiveness of second‐line therapy was suboptimal (< 90%); however, the combination of bismuth‐amoxicillin‐levofloxacin prescribed for 14 days reported successful cure rates. In the third‐line, the classical bismuth‐quadruple therapy with metronidazole‐tetracycline provided acceptable results (87%). Alternatively, dual therapy with vonoprazan and amoxicillin and rifabutin‐based bismuth‐quadruple therapy showed promising results in third‐ and fifth‐line rescue treatment.

## 1 Introduction

Although recent studies have suggested that the prevalence of *Helicobacter pylori* ( *H. pylori* ) infection has decreased in some regions of Brazil, approximately 50% of the population is still infected [1]. This infection is considered the main etiological agent of peptic ulcer, gastric adenocarcinoma, and gastric mucosa‐associated lymphoid tissue (MALT) lymphoma [2]. Its eradication promotes the healing of ulcerative lesions and active chronic gastritis and reduces the risk of stomach cancer in infected individuals [3].

Although *H. pylori* was discovered in the 1980s, the ideal treatment is still not well established. Some authors advocate that only regimens with excellent eradication rates (≥ 95%) should be prescribed, although regimens with eradication rates above 90% are considered good [4]. Treatment failure can be associated with different conditions, such as high bacterial density, infections with cytotoxin‐associated gene‐positive strains, and immunodeficiency disorders [5]. However, these conditions are less relevant than poor adherence to treatment and antimicrobial resistance [6, 7]. Strategies to optimize empirical treatment include extending treatment to 14 days, adding bismuth, and increasing acid inhibition [8].

Given the growing concern about *H. pylori* resistance to antimicrobials, different global consensuses recommend validating therapeutic regimens regionally. In 2018, the IV Brazilian Consensus on *H. pylori* infection recommended extending treatment to 14 days, especially in clarithromycin‐based triple therapy, to obtain better results [9]. If triple therapy fails, the consensus recommends levofloxacin‐based triple therapy or a bismuth‐based quadruple therapy for 10–14 days. However, third‐, fourth‐ and fifth‐line salvage therapies are sometimes necessary since eradication rates decline with retreatment regimens [10]. Although eradication rates below 90% are considered unacceptable for an infectious disease, the effectiveness of second‐line regimens offers an average 80% eradication rate, a frequency also found in a previous Brazilian study [11]. Third‐line regimens without optimization have even lower rates (70%) [12], although no Brazilian studies have yet been carried out on the effectiveness of treatments after the second line.

Since *H. pylori* susceptibility tests are generally unavailable worldwide [9], an alternative is to assess bacterial resistance and optimize *H. pylori* eradication rates through antimicrobial stewardship programs proposed in the management of *H. pylori* infection [13].

This prospective, noninterventional, real‐life study aimed to assess the retreatment regimens empirically prescribed by Brazilian gastroenterologists in the five regions of Brazil to adult patients infected with *H. pylori* and with at least one eradication failure, considering effectiveness, adherence, and adverse effects.

## 2 Methods

### 2.1 Brazilian Registry on *Helicobacter pylori* Management (Hp‐BrazilReg)

The Brazilian Registry on *H. pylori* management (Hp‐BrazilReg) is a multicenter, prospective, noninterventionist study conducted in partnership with the European Registry on *H. pylori* management (Hp‐EuReg) [14]. Both registries are integral components of the Worldwide registry on *H. pylori* management (WorldHpReg). The Hp‐BrazilReg, was launched in March 2022 following approval of the local Ethics Committee (approval number CAAE 52595321.0.0000.5149). Initial findings on first‐line empirical treatment have recently been published [15].

### 2.2 Participants

Variables and outcomes were recorded using an electronic case report form (e‐CRF) provided by the collaborative research platform REDCap hosted at “Asociación Española de Gastroenterología” (AEG; www.aegastro.es), a nonprofit scientific and medical society focused on gastroenterology research [16]. Data were anonymised and the following variables were recorded: patients' demographics, any previous eradication attempts, treatments used, compliance and effectiveness, as well as safety outcomes. Further information on the variables is available in the published protocol [14]. Written, informed consent was obtained from all patients prior to study entry.

### 2.3 Data Management

All cases filed until October 2024 regarding retreatment (second‐, third‐, fourth‐, and fifth‐line therapies or more) were included for analysis. The database cohort was reviewed for inconsistencies and subsequent data cleaning. Systematic monitoring was performed for data coherence on a routine basis (detailed information is summarized in Appendix S1).

### 2.4 Statistical Analysis

#### 2.4.1 Categorization and Definition of Variables

In addition to the therapeutic antibiotics/antimicrobials prescribed, their doses and intakes as well as the duration of treatment were assessed using the three most frequent categories: 7, 10, and 14 days.

The potency of proton‐pump inhibitors (PPIs) was assessed as defined by Graham et al. [17] and Kirchheiner [18]: low dose (between 4.5 and 27 mg of omeprazole equivalent twice daily), standard dose (between 32 and 40 mg of omeprazole equivalent twice daily), and high dose (between 54 and 128 mg of omeprazole equivalent twice daily). When vonoprazan (VPZ) was used, the dose and number of doses per day were recorded. The use or lack of probiotics with antimicrobial treatment was also recorded.

Effectiveness was investigated by modified intention‐to‐treat (mITT), analyzing all patients who completed the follow‐up (eradication control test after treatment with a result of success or failure), regardless of adherence [14]. mITT was defined as the main effectiveness analysis since it most closely reflects the results of clinical practice [19].

Good adherence to treatment was defined as having used at least 90% of the prescribed regimen. Adverse events were assessed at each patient's follow‐up visits.

#### 2.4.2 Data Analysis

Continuous variables were expressed as mean ± standard deviation (SD). Qualitative variables were expressed as absolute or relative frequencies with percentages (%). Differences between groups were determined using the chi‐square test or then Fisher's exact test, when appropriate. The results were considered significant if *p* < 0.05.

Multivariate analysis consisted of a logistic regression model used to assess factors intervening in effectiveness, with mITT (success) as the dependent variable. The independent factors evaluated were age, gender, treatment indication, treatment duration (7, 10, and 14 days), PPI dose (low, standard, and high), VPZ use, adherence (no: below 90% vs. yes: above 90%), use of bismuth, repetition of drugs used in previous treatments. Multivariate analyses compared regions of the country and different regimens in the second‐line treatment but were not performed for other lines due to the heterogeneity of regimens and limited sample size in these regions. Odds ratios and 95% confidence intervals were reported. The quality of the residuals of the logistic regression model was analyzed.

## 3 Results

**FIGURE 1:**
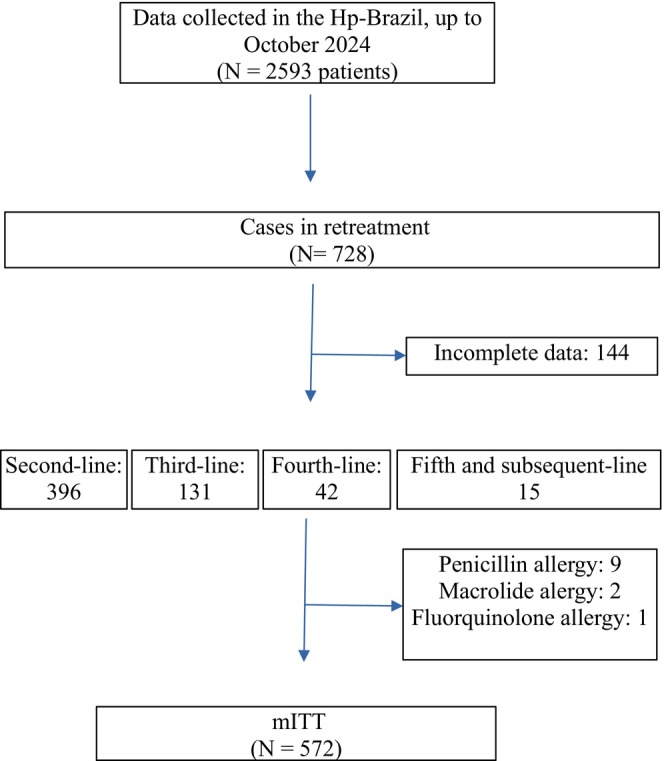
Study flow chart. Hp‐Brazil Reg; mITT, modified intention‐to‐treat.

Data on 728 retreatment cases in Brazil dated from March 2022 to October 2024 were extracted from the Hp‐BrazilReg study. Among those, 144 cases were excluded for incomplete data that prevented the calculation of mITT, mainly due to the lack of an eradication control test and information on the type of eradication regimen used. Another 12 patients were excluded from the analysis for their allergy to antibiotics, which limits the choice of medications and does not represent the general population (Figure 1). Due to the small number of cases in the sixth‐line treatment (*n* = 2), data from fifth‐ and sixth‐line treatments were pooled and analyzed together as fifth‐line or more. The baseline characteristics of the 572 patients included in the study are shown in Table 1.

**TABLE 1:** Baseline characteristics of overall 572 patients included in the study.

Variable	Brazil	South‐Eastern	Southern	North‐Eastern	Northern	Central‐Western
Number of cases, *n* (%)	572 (100%)	239 (41.8%)	124 (21.7%)	94 (16.4%)	79 (13.8%)	36 (6.3%)
Female, *n* (%)	366 (64%)	161 (67%)	69 (55.6%)	51 (41.1%)	62 (78%)	23 (63.8%)
Age, mean (SD)	52.5 (13.3)	54.5 (13.9)	49 (14.3)	55 (11.6)	51.5 (11.6)	48.9 (13.2)
Indication for investigation of infection, *n* (%)						
Dyspepsia	370 (64.6%)	133 (56%)	80 (64.5%)	84 (89%)	45 (57%)	28 (77.7%)
Ulcer	53 (9.2%)	26 (10.8%)	11 (8.8%)	5 (5.3%)	6 (7.5%)	5 (13.8%)
Pre‐neoplastic lesions	31 (5.4%)	24 (10%)	3 (2.4%)	2 (2%)	—	2 (5.4%)
Others	118 (20.6%)	56 (23.4%)	30 (24.2%)	3 (3.2%)	28 (35.4%)	1 (2.7%)
Treatment regimens, *n* (%)						
Triple	327 (57.2%)	136 (57%)	60 (48.4%)	58 (61.7%)	50 (63%)	23 (63.8%)
Quadruple	178 (31.1%)	88 (36.8%)	49 (39.5%)	12 (12.7%)	20 (25%)	9 (25%)
Dual	63 (11%)	12 (5%)	14 (11.3%)	24 (25.5%)	9 (11.4%)	4 (11.1%)
Three‐in‐one single‐capsule^a^	3 (0.5%)	3 (1.2%)				
Quintuple	1 (0.2%)		1 (0.8%)			
Bismuth‐containing therapies, *n* (%)	178 (31.1%)	86 (35.9%)	51 (41.1%)	12 (12.7%)	20 (25.3%)	9 (25%)
Duration of treatment, *n* (%)						
6 days	1 (0.2%)	1 (0.4%)	—	—		
7 days	13 (2.3%)	1 (0.4%)	12 (9.7%)	—		
10 days	118 (20.6%)	22 (9.2%)	12 (9.7%)	40 (42.6%)	28 (35.5%)	16 (44.4%)
14 days	434 (75.9%)	210 (88%)	100 (80.6%)	54 (57.4%)	51 (64.5%)	20 (55.6%)
15 days	3 (0.5%)	3 (1.3%)	—	—		
21 days	2 (0.3%)	2 (0.8%)	—	—		
Duration of treatment in days	13 (1.94)	13.3 (1.75)	12.9 (2.28)	12.3 (1.99)	13 (1.77)	12.2 (2.02)
Median (SD)						
Dose PPI, *n* (%)	424 (74.3%)	201	61	60	70	32
Low^b^	228 (39.8%)	77 (32.2%)	24 (19%)	41 (43.6%)	67 (84.8%)	19 (52.7%)
Standard	59 (10.3%)	29 (12.1%)	29 (23.3%)	1 (1%)	—	—
High	136 (23.8%)	94 (39.3%)	8 (6.5%)	18 (19%)	3 (3.7%)	13 (36.1%)
Unknown	1 (0.2%)	1 (0.4%)				
Dose PCAB^c^, *n* (%)	147 (25.7%)	37 (15.5%)	63 (50.8%)	34 (36.1%)	9 (11.4%)	4 (11.1%)
Compliance, *n* (%)						
No (< 90% drug intake)	6 (1%)	4 (0.6%)	2 (0.4%)	—	—	—
Yes (≥ 90% drug intake)	566 (98.9%)	235	122	94 (100%)	79 (100%)	36 (100%)
		(98.3%)	(98.4%)			

^a^ Three‐in‐one single capsule containing bismuth, tetracycline, and metronidazole.

^b^ Low‐dose PPI: 4.5–27 mg omeprazole equivalents, two times per day (e.g., 20 mg omeprazole equivalents, two times per day). Standard‐dose PPI: 32–40 mg omeprazole equivalents, two times per day (e.g., 40 mg omeprazole equivalents, two times per day). High‐dose PPI: 54–128 mg omeprazole equivalents, two times per day (e.g., 60 mg omeprazole equivalents, two times per day).

^c^ PCAB: vonoprazan 20 mg, twice daily.

The mean age of patients was 52.5 ± 14.3 years, and 64% were female. Most patients (93.3%) were diagnosed with infection by an invasive method (histology following specimen collection during upper endoscopy), and 3% underwent carbon‐labeled urea breath tests [carbon14‐labeled urea breath test (^14^C‐UBT) in 2.8% and carbon13‐labeled urea breath test (^13^C‐UBT) in 0.2%]. As for the eradication control test, histology was used in 89% of cases, ^13^C‐UBT or ^14^C‐UBT in 9%, and fecal antigen testing in 2%.

### 3.1 Prescriptions

Among the 572 cases analyzed, 386 (67.5%), 130 (22.7%), 42 (7.3%), and 14 (2.4%) cases corresponded to prescriptions for second‐, third‐, fourth‐, or fifth‐line or more treatments, respectively. Those 572 cases were distributed across the five regions of the country: South‐Eastern (239, 41.8%), Southern (124, 21.7%), Northeastern (94, 16.4%), Northern (79, 13.8%), and Central‐West (36, 6.3%).

Treatment lasted a median of 14 days. PPIs were used in 74% of the regimens, and VPZ in 26%.

In second‐line, the most used regimen was the association of PPI + amoxicillin + levofloxacin for 10–14 days (55%), PPI + clarithromycin + amoxicillin (9%), and PPI + bismuth + amoxicillin + levofloxacin (8%). In the third‐line, the regimen containing PPI‐bismuth + tetracycline + metronidazole (24%) was the most used, followed by PPI + bismuth + amoxicillin + doxycycline (15%) and dual vonoprazan + amoxicillin (10%). In the fourth‐line, amoxicillin‐VPZ dual therapy was the most used regimen (33.3%), followed by quadruple PPI + bismuth + amoxicillin + rifabutin (14.3%). Dual therapy with VPZ + amoxicillin was also the most used regimen as fifth‐line or more in 43% of cases. The overall therapies used in different lines are reported in Tables S1–S4.

A total of 97 patients (18%) used probiotics, with 13% in second‐line treatments, 4% in third‐line, 0.5% in fourth‐line, and 0.5% in fifth‐line or more.

### 3.2 Effectiveness of Second‐Line Treatments

Second‐line treatments were prescribed in 386 patients. The overall effectiveness of second‐line treatments was 74%. The overall triple therapy with PPI + levofloxacin + amoxicillin showed 66% eradication rate with eradication rate of 84% during 14 days and 55% during 10 days. The 14‐day therapy with PPI + bismuth + levofloxacin + amoxicillin achieved 100% eradication rate, while the 14‐day therapy with VPZ + levofloxacin + amoxicillin had 88.9% eradication rate. The classic 14‐day triple PPI + clarithromycin + amoxicillin and the 14‐day double therapy with VPZ‐amoxicillin had a 75.7% and 75% eradication rate, respectively.

Quadruple therapy with PPI + bismuth + levofloxacin + amoxicillin was superior to the triple therapy with PPI + levofloxacin + amoxicillin (*p* = 0.016, OR 6.2; 95% CI 1.40–27.3), even when only 14‐day regimens were compared. The differences in effectiveness between the PPI + levofloxacin + amoxicillin and VPZ + levofloxacin + amoxicillin were not statistically significant (*p* = 0.06). The mITT effectiveness of the most commonly prescribed therapies by second‐line treatment regimens are described in Table 2.

**TABLE 2:** Effectiveness by modified intention‐to‐treat of the most commonly prescribed therapies by second‐line treatment.

Second‐line		Brazil			South‐Eastern			Southern			North‐Eastern			Northern			Central‐Western		
		Use, *N*	mITT, *N*	% (95% CI)	Use, *N*	mITT, *N*	% (95% CI)	Use, *N*	mITT, *N*	% (95% CI)	Use, *N*	mITT, *N*	% (95% CI)	Use, *N*	mITT, *N*	% (95%, CI)	Use, *N*	mITT, *N*	% (95%, CI)
A + L + PPI		212	139	65.6 (59–72)	85	60	70.6 (60–80)	18	12	66.7 (41–87)	38	23	60.5 (43–76)	49	34	69.4 (55–82)	22	10	45.5 (25–68)
	14 days	115	84	73 (64–81)	77	54	70 (59–80)	8	6	75 (35–97)	3	3	100 (29–100)	21	18	85.7 (64–97)	6	3	50 (12–88)
	10 days	97	55	56.7 (46–67)	8	6	75 (35–97)	10	6	60 (26–88)	35	20	57.1 (39–74)	28	16	57.1 (37–75)	16	7	43.8 (20–70)
C + A + PPI		37	28	75.7 (59–88)	23	16	69.6 (47–87)	12	10	83.3 (52–98)	1	1	100 (2.5–100)	—	—	—	1	1	100 (2.5–100)
Bi + A + L + PPI		31	31	100 (88–100)	23	23	100 (85–100)	8	8	100 (63–100)	—	—	—	—	—	—	—	—	—
A + VPZ		24	18	75 (53–90)	6	6	100 (54–100)	13	9	69 (38–90)	4	2	50 (7–93)	—	—	—	1	1	100 (2.5–100)
A + L + VPZ		18	16	88.9 (65–98)	5	5	100 (48–100)	7	5	71.4 (30–96)	6	6	100 (54–100)	—	—	—	—	—	—
Bi + M + Tc + PPI		17	13	76 (50–93)	6	3	50 (12–88)	10	9	90 (56–99)							1	1	100 (2.5–100)
Bi + M + Tc + VPZ		13	12	92.3 (64–100)	1	1	100 (2.5–100)	12	11	91.7 (61–99)	—	—	—	—	—	—	—	—	—
C + A + VPZ		7	6	85.7 (42–99)	—	—	—	3	3	100 (29–100)	4	3	75 (19–99)	—	—	—	—	—	—
Bi + A + L + VPZ		5	4	80 (28–99)	—	—	—	5	4	80 (28–99)	—	—	—	—	—	—	—	—	—
Bi + M + D + PPI		5	5	100 (48–100)	5	5	100 (48–100)	—	—	—	—	—	—	—	—	—	—	—	—
C + L + PPI		4	2	50 (7–93)	—	—	—	—	—	—	4	2	50 (7–93)	—	—	—	—	—	—
Marginal therapies^a^		13	12	92.3	8	7	87.5	3	3	100	2	2	100						
Overall		386	286	73.6 (69–78)	162	126	76.5 (69–82)	91	74	81.3 (72–89)	59	39	66.1 (53–78)	49	34	69.4 (55–82)	25	13	52 (31–72)

Abbreviations: A, amoxicillin; Bi, bismuth; C, clarithromycin; D, doxycycline; L, levofloxacin; M, metronidazole; PPI, proton‐ pump inhibitor; R, rifabutin; Tc, tetracycline hydrochloride; VPZ, vonoprazan.

^a^ Marginal therapies were considered to be those with 3 or fewer cases. There were 11 different regimens, with a total of 13 cases (see Table S1).

### 3.3 Effectiveness of Third‐Line Treatments

The overall effectiveness was 75.4% (Table 3), and the most prescribed regimen, the 14‐day quadruple therapy with PPI + bismuth + tetracycline + metronidazol had 87% eradication rate. The 14‐day therapy with PPI + bismuth + amoxicillin + doxycycline achieved a 65% eradication rate, and the 14‐day double therapy with VPZ + amoxicillin a 100% eradication rate. The 14‐day triple therapy with PPI + clarithromycin + amoxicillin and PPI + levofloxacin + amoxicillin had 71% and 31% eradication rate, respectively. The mITT effectiveness of the most commonly prescribed therapies by third‐line treatment regimens are described in Table 3.

**TABLE 3:** Effectiveness modified intention‐to‐treat of the most commonly prescribed therapies by third‐line treatment.

Third‐line		Brazil			South‐Eastern			Southern			North‐Eastern			Northern			Central‐Western		
		Use, *N*	mITT, *N*	% (95% CI)	Use, *N*	mITT, *N*	% (95% CI)	Use, *N*	mITT, *N*	% (95% CI)	Use, *N*	mITT, *N*	% (95% CI)	Use. *N*	mITT, *N*	% (95%, CI)	Use. *N*	mITT, *N*	% (95%, CI)
Bi + M + Tc + PPI		31	27	87 (70–96)	19	17	89 (67–98)	3	2	67 (9–99)	1	1	100 (2.5–100)	—	—	—	8	7	87 (47–99)
Bi + A + D + PPI		20	13	65 (41–85)	—	—	—	—	—	—	—	—	—	20	13	65 (41–85)	—	—	—
A + VPZ		16	16	100 (80–100)	2	2	100 (16–100)	1	1	100 (2.5–100)	12	12	100 (73–100)	—	—	—	1	1	100 (2.5–100)
A + L + PPI		13	4	31 (9–61)	7	3	43 (18–81)	5	1	20 (0.5–71)	1	1	100 (2.5–100)	—	—	—	—	—	—
	14 days	10	3	30 (7–65)	6	3	50 (12–88)	4	0	0 (−)	—	—	—	—	—	—	—	—	—
	10 days	3	1	33 (8.4–90)	1	0	0 (−)	1	1	(2.5–100)	1	0	0 (−)	—	—	—	—	—	—
Bi + M + D + PPI		10	8	80 (44–97)	4	4	100 (40–100)	—	—	—	6	4	67 (22–95)	—	—	—	—	—	—
Bi + M + Tc + VPZ		10	9	90 (55–99)	4	4	100 (39–100)	6	5	83 (36–99)	—	—	—	—	—	—	—	—	—
C + A + PPI		7	5	71 (29–96)	6	5	83 (36–99)	1	0	0 (−)	—	—	—	—	—	—	—	—	—
Marginal therapies^a^		23	16	69.6	10	7	70	6	4	66.7	3	33.3							
Overall		130	98	75.4 (67–82)	56	46	82 (69–91)	22	13	59 (36–79)	23	18	78 (56–92)	20	13	65 (41–85)	9	8	89 (51–99)

Abbreviations: A, amoxicillin; Bi, bismuth; C, clarithromycin; D, doxycycline; L, levofloxacin; M, metronidazole; PPI, proton‐pump inhibitor; R, rifabutin; Tc, tetracycline hydrochloride; VPZ, vonoprazan.

^a^ Marginal therapies were considered to be those with 3 or fewer cases. There were 17 different regimens, with a total of 23 cases (see Table S1).

### 3.4 Effectiveness of Fourth‐Line Treatments

The fourth‐line treatments had an overall eradication rate of 83%. The 14‐day double therapy with VPZ + amoxicillin achieved a 100% eradication rate, as the 14‐day quadruple therapy with VPZ + bismuth + rifabutin + amoxicillin that presented a 100% eradication rate. The 14‐day therapy with VPZ + levofloxacin + amoxicillin had a 60% eradication rate. The mITT effectiveness of the most commonly prescribed therapies by fourth‐line treatment regimens are described in Table 4.

**TABLE 4:** Effectiveness by modified intention‐to‐treat of the most commonly prescribed therapies by fourth‐line treatment.

	Brazil			South‐Eastern			Southern			North‐Eastern			Northern			Central‐Western		
Fourth‐line	Use, *N*	mITT, *N*	% (95% CI)	Use, *N*	mITT, *N*	% (95% CI)	Use, *N*	mITT, *N*	% (95% CI)	Use, *N*	mITT, *N*	% (95% CI)	Use. *N*	mITT, *N*	% (95%, CI)	Use. *N*	mITT, *N*	% (95%, CI)
A + VPZ	14	14	100 (77–100)	2	2	100 (16–100)	—	—	—	1	1	100 (2.5–100)	9	9	100 (66–100)	2	2	100 (16–100)
Bi + A + R + VPZ	6	6	100 (54–100)	6	6	100 (54–100)	—	—	—	—	—	—	—	—	—	—	—	—
A + L + VPZ	5	3	60 (15–95)	—	—	—	5	3	60 (15–95)	—	—	—	—	—	—	—	—	—
Marginal therapies^a^	17	12	70.6	7	6	85.7	4	4	100	5	1	20	1	1	100			
Overall	42	35	83 (68–93)	15	14	93 (68–99)	9	7	78 (40–97)	6	2	33 (4.3–77)	10	10	100 (69–100)	2	2	100 (16–100)

Abbreviations: A, amoxicillin; Bi, bismuth; L, levofloxacin; R, rifabutin; VPZ, vonoprazan.

^a^ Marginal therapies were considered to be those with 3 or fewer cases. There were 12 different regimens, with a total of 17 cases (see Table S1).

### 3.5 Effectiveness of Fifth‐Line or More Treatments

Among a total of 14 cases (12 on fifth‐ and 2 on sixth‐line), the most common therapy was the 14‐day double therapy with VPZ + amoxicillin, with a 100% eradication rate. The mITT effectiveness of the most commonly prescribed therapies by fifth‐line or more treatment regimens are described in Table 5.

**TABLE 5:** Effectiveness by modified intention‐to‐treat of the most commonly prescribed therapies by fifth‐line treatment or more.

	Brazil			South‐Eastern			Southern			North‐Eastern			Northern			Central‐Western		
Fifth‐line and more	Use, *N*	mITT, *N*	% (95% CI)	Use, *N*	mITT, *N*	% (95% CI)	Use, *N*	mITT, *N*	% (95% CI)	Use, *N*	mITT, *N*	% (95% CI)	Use. *N*	mITT, *N*	% (95%, CI)	Use. *N*	mITT, *N*	% (95%, CI)
A + VPZ	6	6	100 (54–100)	2	2	100 (16–100)	—	—	—	4	4	100 (40–100)	—	—	—	—	—	—
Marginal therapies^a^	8	6	75	4	3	75	2	2	100	2	2	50						
Overall	14	12	85 (57–98)	6	5	83 (36–99)	2	2	100	6	5	83 (36–99)	—	—	—	—	—	—

Abbreviations: A, amoxicillin; VPZ, vonoprazan.

^a^ Marginal therapies were considered to be those with 3 or fewer cases. There were 8 different regimens, with a total of 8 cases (see Table S1).

The Tables S5–S8 show the effectiveness of all empirical prescribed regimens in different lines of retreatment among the five regions of the country.

### 3.6 Safety and Compliance

Treatment adherence occurred in 99% of cases and at least one adverse effect was reported in 23% (Table S9). No serious adverse effects were reported, nor treatment interruption due to adverse effects (Table S10). The most common adverse effects were nausea (14%; mean duration, 6 days), metallic taste (8.4%; mean 6 days), and diarrhea (4.9%; mean 4 days).

### 3.7 Comparative and Multivariate Analysis

Table 6 summarizes the statistical analysis results. Univariate analysis showed that therapy with levofloxacin, amoxicillin, and PPI was less effective in the Central‐West region of the country (OR 2.9; 95% CI: 1.10–7.5; *p* = 0.031) compared to the Southeast region. This difference was not significant in the other regions.

**TABLE 6:** Statistical analysis effectiveness in rescue therapy in Brazil.

Independent variables	OR (95% CI)	*p*
*All retreatment groups*		
Age (mean)		
Cured (53.5 years) × Failed (49.6 years)	1.02 (1.008–1.039)	0.0393
Duration of treatment (reference 10 days)		
14 days (79%) × 10 days (60%)	1.8 (1.2–2.9)	0.007
Coadjuvant (reference vonoprazan)		
VPZ (88%) × PPI (77%)	2.87 (1.69–5.13)	0.0002
VPZ (88%) × high PPI (73%)	3.03 (1.5–7.3)	0.003
VPZ (88%) × low PPI (65%)	3.4 (1.5–7.6)	0.003
Use bismuth × no bismuth	1.8 (1.10–2.9)	0.0196
Bismuth‐quadruple therapy × triple therapy (no bismuth)	2.7 (1.7–4.3)	< 0.001
Other statistical analysis		
A + L‐PPI 14 days × A + L‐PPI 10 days	1.84 (1.06–3.2)	0.029
B + A + L‐PPI 14 days × A + L‐PPI 14 days	6.2 (1.40–27.3)	0.016
A‐VPZ × A + L‐PPI 14 days	3.8 (1.5–9.7)	0.004
Second‐line of treatment		
South‐Eastern × Central‐Western	3.0 (1.3–7.2)	0.012
A + L‐PPI in South‐Eastern × A + L‐PPI in Central‐Western	2.9 (1.10–7.5)	0.031
Probiotic × adverse effects		
Adverse events (reference no use of probiotic)	4.24 (2.7–6.8)	< 0.01

*Note:* Treatment success was defined as mITT eradication. Low‐dose PPI: 4.5–27 mg omeprazole equivalents, two times per day (e.g., 20 mg omeprazole equivalents, two times per day). Standard‐dose PPI: 32–40 mg omeprazole equivalents, two times per day (e.g., 40 mg omeprazole equivalents, two times per day). High‐Dose PPI: 54–128 mg omeprazole equivalents, two times per day (e.g., 60 mg omeprazole equivalents, two times per day).

Abbreviations: A, amoxicillin; B, bismuth; D, doxycycline; L, levofloxacin; M, metronidazole; PPI, proton‐pump inhibitor; Tc, tetracycline hydrochloride; VPZ, vonoprazan.

Multivariate analysis revealed that the only independent factors associated with treatment effectiveness were 14‐day treatment duration (OR 1.8; 95% CI: 1.2–2.9), age (OR 1.02; 95% CI: 1.008–1.039), use of bismuth (OR 1.78; 95% CI: 1.10–2.93), and use of VPZ (OR 2.87; 95% CI 1.69–5.13).

Quality analysis of residuals showed that the logistic regression model was adequate (Figure S1). The 14‐day regimens had higher eradication than the 10‐day regimens [(79% × 60%, respectively); *p* = 0.007, OR: 1.8, 95% CI: 1.2–2.9]. The mean age of cured patients (53.5 years) was statistically higher than that of patients who failed eradication (49.6 years). No differences were found in *H. pylori* eradication in relation to sex or treatment indication (dyspepsia versus ulcer).

The use of bismuth had superior eradication rates compared to regimens that did not use it [(84% vs. 70%, respectively); *p* = 0.0196, OR: 1.8, 95% CI: 1.10–2.9]. Quadruple regimens with bismuth were superior to triple regimens without bismuth (*p* < 0.001, OR: 2.7, 95% CI: 1.7–4.3). However, this difference was not found (*p* = 0.8) when comparing quadruple regimens with bismuth with double regimens.

The eradication rates of PPI‐containing regimens were statistically lower than those with VPZ‐containing regimens [(77% vs. 88%), respectively; *p* = 0.0002, OR: 2.87, 95% CI: 1.69–5.13]. This difference between VPZ and PPI‐regimens was observed in low‐dose PPI‐containing regimens (*p* = 0.003, OR: 3.4, 95% CI: 1.5–7.6) and in high‐dose PPI‐containing regimens (*p* = 0.003, OR: 3.3, 95% CI: 1.5–7.3).

Among PPI‐containing regimens, no differences in effectiveness were found in relation to the dose of PPI used (high, low, or standard dose).

No differences were found between regimens with bismuth, metronidazole, tetracycline, and PPI and regimens with bismuth, metronidazole, doxycycline, and PPI in relation to the eradication rate.

Considering all retreatment lines, the 14‐day therapy with levofloxacin, amoxicillin, and PPI was superior to that used for 10 days. The quadruple therapy with bismuth, levofloxacin, amoxicillin, and PPI was superior to the triple therapy with levofloxacin, amoxicillin, and PPI. Moreover, the double therapy with amoxicillin and VPZ was superior to the triple therapy with levofloxacin, amoxicillin, and PPI.

The second‐line treatment sub‐analysis showed that the Southeast region had better results than the Central‐West due to the better performance of the therapy with levofloxacin, amoxicillin, and PPI.

The use of probiotics was significantly associated with a reduction in adverse events (18.4% vs. 49%; OR 4.24; 95% CI 2.7–6.8).

## 4 Discussion

Our study analyzed 572 cases of patients undergoing multiple treatments: 386 with second‐line and 186 with rescue treatments. The overall eradication rate for second‐line therapy was 74%, with the PPI + amoxicillin + levofloxacin regimen (10–14 days) used by 55% of patients, achieving 84% effectiveness (14 days) and 55% (10 days). Adding bismuth to the 14‐day regimen increased effectiveness to 100%. In third‐line treatment, the PPI‐bismuth‐tetracycline‐metronidazole regimen was used by 24% of cases, with 87% eradication. The fourth‐line treatment commonly used was amoxicillin‐VPZ dual therapy (33%), followed by a quadruple regimen showing 100% effectiveness.

Cured patients had a higher mean age than those who failed retreatment. Studies indicate that older patients tend to have better *H. pylori* eradication rates, potentially due to factors like gastric atrophy [20, 21]. However, a recent European study found no significant differences in effectiveness between age groups [22]. Our study also highlighted the extensive use of endoscopy for initial diagnosis (93.3%) and cure control tests (89%), reflecting the limited availability of noninvasive methods in Brazil. Similar findings were noted in two European multicenter studies, suggesting a need to optimize noninvasive methods for young dyspeptic patients at low risk for gastric disease [23, 24].

### 4.1 Treatment Effectiveness

#### 4.1.1 Second‐Line Treatments

The overall eradication rate of second‐line treatments corresponded to a mITT of 74%. The most common regimen (55%) was the triple therapy with amoxicillin, levofloxacin, and PPI, showing a significantly greater effectiveness when used for 14 days. Although a 10‐day treatment is available in Brazil, the results show a low effectiveness of this therapy, even when used for 14 days. When analyzing second‐line treatments in the Hp‐EuReg, Nyssen et al. [25] found an overall effectiveness of 84%, with the therapy with levofloxacin, amoxicillin, and PPI as the most prescribed and with an 81% eradication rate. However, in our study, patients who were treated with the 14‐day therapy with amoxicillin, levofloxacin, and VPZ eradicated mITT in 88.9% of cases. VPZ was also used as a second‐line alternative by 14 days in dual therapy with amoxicillin, with a mITT of 75%. Further studies are required to determine whether these results do not depend only on the increased effectiveness of amoxicillin promoted by VPZ [26].

The addition of bismuth to the 14‐day triple therapy with levofloxacin was used in 11% of cases and was effective with 100% mITT, consistent with studies reported in Asia [27] and Europe [25]. It should be noted that bismuth salts are not commercially available in Brazil, either alone or in a three‐in‐one capsule containing bismuth, tetracycline, and metronidazole [28], and they are only available through manipulation. In our study, 14‐day quadruple therapy with bismuth, tetracycline, and metronidazole was used in 7.8% of cases, associated with PPI in 4.5% and VPZ in 3.3%, with mITT eradication rates of 76%. Further studies with a larger number of patients are required to confirm the superiority of adjuvant VPZ over PPI in quadruple therapy, since this therapy is largely independent of marked acid suppression, although metronidazole‐resistant strains respond better to PPI [29]. Bismuth compounds have multiple antibacterial effects through mechanisms that are still poorly understood [30] but apparently exert additive effects with antibiotics [31, 32, 33].

#### 4.1.2 Third‐Line Treatments

The overall effectiveness of third‐line treatments (75.4%) was like that found in Europe by Burgos‐Santamaría (79%) [12]. The most effective regimens were quadruple therapy with bismuth, metronidazole, tetracycline, and PPI and double therapy with amoxicillin and VPZ, with mITT eradication rates of 87% and 100%, respectively. The use of VPZ as an alternative to PPI in quadruple therapy with bismuth, metronidazole, and tetracycline had a 90% eradication rate. Replacing tetracycline with doxycycline in quadruple therapy did not show any significant difference in terms of effectiveness. In addition to these, a wide variety of empirical regimens were found with eradication rates close to 70%, confirming previous European studies that also show heterogeneity in the management of the infection [12, 34].

#### 4.1.3 Fourth and Fifth‐Line and More Treatments

Fourth and fifth‐line or more treatments had fewer cases than previous lines and showed mITT eradication rates of 83%. As for fourth‐line treatments, the most used and effective regimens were double therapy with amoxicillin and VPZ (40%) and bismuth, amoxicillin, rifabutin, and VPZ (17%), both with mITT eradication rates of 100%. Regarding fifth‐line or more treatments, the most used and effective regimen was double therapy with amoxicillin and VPZ (50%), with an eradication rate of 100%. A significant part of the good results obtained with these salvage treatments can be linked to the use of VPZ as an adjuvant, confirming Asian studies from a recent meta‐analysis [35]. The superiority of VPZ has been attributed to its rapid, potent, and stable acid‐suppressing action, which promotes the elevation of intragastric pH, inducing the replicative phase of the bacteria and maximizing the action of amoxicillin [36]. In addition, the pharmacokinetic characteristics of VPZ are not impacted by diet and polymorphism of cytochrome CYP2C19. Since *H. pylori* resistance to amoxicillin is low, the potent acid inhibition promoted by VPZ optimizes eradication treatment [37].

#### 4.1.4 Dual Therapy

The 14‐day double therapy with amoxicillin and VPZ was used in the retreatment of 60 patients (*n* = 24 in second‐line, *n* = 16 in third‐line, *n* = 14 in fourth‐line, and *n* = 6 in fifth‐line or more treatments). The dose of amoxicillin ranged from 2 – 4 g per day in 2–4 doses. Among all patients, 97% received 3–4 doses per day, with no differences in eradication rates. Similarly, no significant differences were found in relation to doses of 3 g or 4 g per day (Figure S2). In a recent study assessing different doses of amoxicillin in first‐line double therapy with VPZ, Hu et al. [38] found no significant differences in treatment effectiveness in relation to doses of 2 g or 3 g per day. Further studies are required to define the optimal dose of amoxicillin and the optimal number of doses per day in dual therapy with amoxicillin and VPZ.

#### 4.1.5 Rifabutin‐Based Therapies

Promising results were also found in third‐ and fourth‐line treatments with quadruple regimens with bismuth, amoxicillin, and rifabutin, with 100% effectiveness. The in‐vitro sensitivity of *H. pylori* to rifabutin is high and this antibiotic has a lower minimum inhibitory concentration than amoxicillin, clarithromycin, and metronidazole [39]. Gisbert et al. [40] found that the mean resistance rate of *H. pylori* to rifabutin was 0.13%. However, this antibiotic is not available in Brazil, and its use requires importation [9]. A previous study reported that regimens containing rifabutin had a mean eradication rate of 73.5%, specifically 73% in first‐line treatments, 78% second‐line, 80% in third‐line, and 66% in fourth‐line [41]. However, when used in a quadruple regimen with bismuth, rifabutin can lead to rates up to 30% higher [42], which can explain the higher eradication rates found in our study in relation to those observed in the review by Gisbert et al. [40]. However, our sample is small, and further studies are required to better understand this regimen.

#### 4.1.6 PPI and VPZ as Adjuvant

High doses of PPI were used in 24% of cases in our study, with a standard dose in 10% and low doses in 40%. In our country, the habit of prescribing commercial kits with low doses of PPIs possibly favored the prescription of low doses. However, the PPI dose was not an independent variable in relation to effectiveness. Our results agree with those described by Wang et al. [43] in a meta‐analysis that compared different doses of esomeprazole with omeprazole or pantoprazole without finding significant differences.

Vonoprazan was used in 26% of cases in doses of 20 mg twice daily, although its use is not yet approved in Brazil as adjuvant in *H. pylori* treatment. Multivariate analysis showed that overall VPZ use was an independent factor to eradication compared to PPI [(88% × 77%, respectively); *p* = 0.0002, OR: 2.87, 95% CI: 1.69–5.13].

#### 4.1.7 Probiotics

The use of probiotics was associated with a significant reduction in adverse effects derived from *H. pylori* treatment. Our study did not consider the type of probiotic used, the dose, and the number of doses, so no further conclusions can be made. Its use has not yet been established, and can be individualized, as proposed by Buzás et al. [44] in a study that recommends its use for elderly patients with comorbidities, individuals with a previous history of antibiotic‐induced diarrhea, or individuals who had other adverse effects in previous *H. pylori* treatments, as well as at the patient's request.

### 4.2 Adherence and Adverse Effects

Adherence to the prescribed regimens was high (99%) and at least one adverse effect was reported in 23.4% of cases, with no serious adverse effects nor effects requiring treatment discontinuation. Adverse effects did not significantly interfere with treatment adherence, as also observed by Nyssen et al. [45] when analyzing side effects of *H. pylori* treatments in more than 22,000 patients.

### 4.3 Results in Different Regions of Brazil

Regarding effectiveness differences in the different regions of Brazil, for second‐line treatments, the triple therapy with levofloxacin was more effective in the Southeast region than in the Central‐West region. This can be related to the greater resistance to levofloxacin in the Central‐West region, as shown in a national study published in 2016 that found primary resistance to levofloxacin in 15.4% of the Central‐West region compared to 13.8% in the Southeast region [7]. Despite the size of the country and the socioeconomic differences between the different regions of Brazil, no other significant differences were found in effectiveness for the regimens used in the different regions.

### 4.4 Limitations and Strengths of the Study

The main limitation of our study comes from its observational design, lacking randomization and intervention, which may lead to selection bias and heterogeneous results that do not equally represent the studied regions. This non‐randomized approach necessitates caution in interpreting the effectiveness of different regimens. The involvement of gastroenterologists as recruiters may also influence findings, as results could differ if general practitioners were involved. The variety of treatments, distributed across numerous regimens, complicates statistical analysis and limits sub‐analyses regarding PPI dosage, treatment duration, and comparative effectiveness. Despite these challenges, the study encompasses over 570 real‐life cases managed by gastroenterologists across Brazil, reflecting clinical practice. Additionally, the use of the WorldHpReg platform for data entry and monitoring enhances the study's credibility through real‐time quality control, thereby strengthening our findings [14].

## 5 Conclusions

Retreatment of *H. pylori* infections in Brazil shows great heterogeneity among the empirical treatments used. The overall effectiveness of second‐line therapy showed suboptimal (< 90%) cure rate. The 14‐day therapy with amoxicillin, levofloxacin, and PPI was the most widely used as a second‐line treatment, with results considered globally unacceptable (below 80%). However, the combination of bismuth‐amoxicillin‐levofloxacin prescribed for 14 days reported successful effectiveness. As a third‐line treatment, quadruple therapy with bismuth, tetracycline, metronidazole, and PPI had acceptable results. As fourth‐line treatment, schemes with VPZ + bismuth + rifabutin + amoxicillin and dual therapy with VPZ + amoxicillin were the most effective treatments. Dual therapy VPZ + amoxicillin was the most effective in fifth‐line or more treatment. Further prospective studies are required to confirm our findings and subsequently provide and update guidelines for the *H. pylori* retreatment in Brazil.

## Author Contributions

Bruno S. F. Sanches and Luiz Gonzaga V. Coelho planned and coordinated the study. Sandro R. Chaves performed the data extraction. Jussiane N. Gonçalves analyzed, summarized and interpreted the data. All authors revised and approved the final submission. Bruno S. F. Sanches, Sandro R. Chaves, Julio S. Veloso, Leonardo S. Silva, Jame R. Marinho, Hoiti Okamoto, Gustavo C. Couto, Helenice P. Breyer, Christiane S. Alencar, Ernesto Comelli, Luis A. S. Sousa, Mariana Horn, Maria J. G. Massote, Marcela T. R. Loures, Maria F. P. Vidal, Rosival V. Paula, Laercio T. Ribei, Humberto O. Galizzi, Daniel A. A. Terra, Guilherme G. L. Cançado, Bibiana P. Burmann, Jardel S. Caetano, Luiz F. Pena, Maria A. Decanio, Heitor S. Souza, Aline S. O. Kuniyoshi, Ludmila R. Guedes, Maria C. F. Passos, Frederico P. Marinho, Isadora Z. Bombassaro, Aline G. Domingues, Jaques G. Barbosa, Islaine M. Nogueira, Ana F. P. Ramos, Daniella R. Korman, Teogenes B. Souza, Moni C. Barbosa, Decio Chinzon, Leandro L. Silva, Augusto Mantovani, Alzimara H. A. Freitas, Christiane. S. Poncinelli, Marcelo A. Francato collected data, critically reviewed the various drafts of the manuscript, and approved the final submission. Anna Cano and Pablo Parra performed the monitoring and quality check of the data, and approved the final submission. Olga P Nyssen, Hp‐EuReg and WorldHpReg Scientific Director, supervised the monitoring and the quality check, assisted with data analysis, interpretation and synthesis, critically reviewed the various drafts of the manuscript and approved the final submission. Anna Cano‐Català, Leticia Moreira, Pablo Parra, Olga P. Nyssen, Javier P. Gisbert are all members of the Hp‐EuReg and WorldHpReg Scientific Committee; critically reviewed the various drafts of the manuscript, and approved the final submission. Javier P. Gisbert, principal investigator of the registry, directed the project, obtained funding, designed the protocol, critically reviewed the various drafts of the manuscript, and approved the final submission. Luiz Gonzaga Vaz Coelho, guarantor of the study.

## Disclosure

Patient and Public Involvement: Patients and/or the public were not involved in the design, or conduct, or reporting or dissemination plans of this research.

## Ethics Statement

Institutional Review Board Statement: This protocol was approved by the Ethics Committee Universidade Federal de Minas Gerais, Belo Horizonte, Brazil (Approval number CAAE 52595321.0.0000.5149), which acted as a Brazilian reference Institutional Review Board.

## Conflicts of Interest

Luiz Gonzaga V. Coelho has served as speaker for Takeda and EMS (Brazil). Javier P. Gisbert has served as speaker, consultant, and advisory member for or has received research funding from Mayoly Spindler, Allergan, Diasorin, Richen, Biocodex and Juvisé. Olga P. Nyssen has served as speaker or has received research funding from Allergan, Mayoly Spindler, Richen, Biocodex and Juvisé. The remaining authors declare no conflicts of interest.

## Supporting information


**Figures S1–S2:** hel70077‐sup‐0001‐AppendixS1.docx.

**Appendix S1:** hel70077‐sup‐0002‐AppendixS2.docx.

## Data Availability

Raw data were generated at AEG‐REDCap. All data relevant to the study are included in the article or uploaded as Appendix S1. De‐identified raw data referring to this study are available from the WorldHpReg and *H. pylori* ‐BrazilReg. Individual participant data will not be shared.
