# Supplementary material for: Empirical Rescue Eradication Therapy for Helicobacter pylori Infection in Second and Subsequent Treatment Lines: Experience From 500 Cases of the Brazilian Registry on H. pylori Management (Hp‐BrazilReg)
Source: Helicobacter. 2025 Oct 14;30(5):e70077. doi: 10.1111/hel.70077 (PMC12521799; doi:10.1111/hel.70077)

**Figure S1. Residual analysis in the logistical regression model to evaluate the effectiveness of *Helicobacter pylori* treatment**


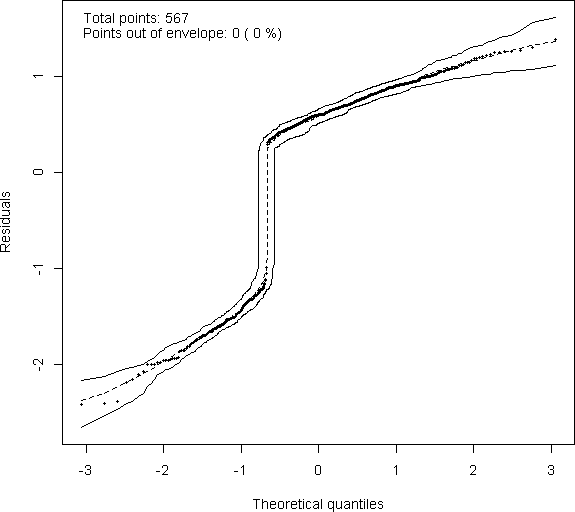


**Figure S2– Daily dosing (a) and times-daily doses of amoxicillin (b) in dual therapy with vonoprazan (N=60)**

Figure S1. Residual analysis in the logistic regression model to evaluate the effectiveness of Helicobacter pylori treatment show the model is adequate

1. As only 2 cases used 2g of amoxicillin/day and one case used 3,5g/day, the comparison between different doses of amoxicillin evaluated 4g/day (N=29) versus 3g/day (N=28).

Comparison of effectiveness between schemes dual amoxicillin-VPZ (reference 3g of amoxicillin):

4g: p= 0.373


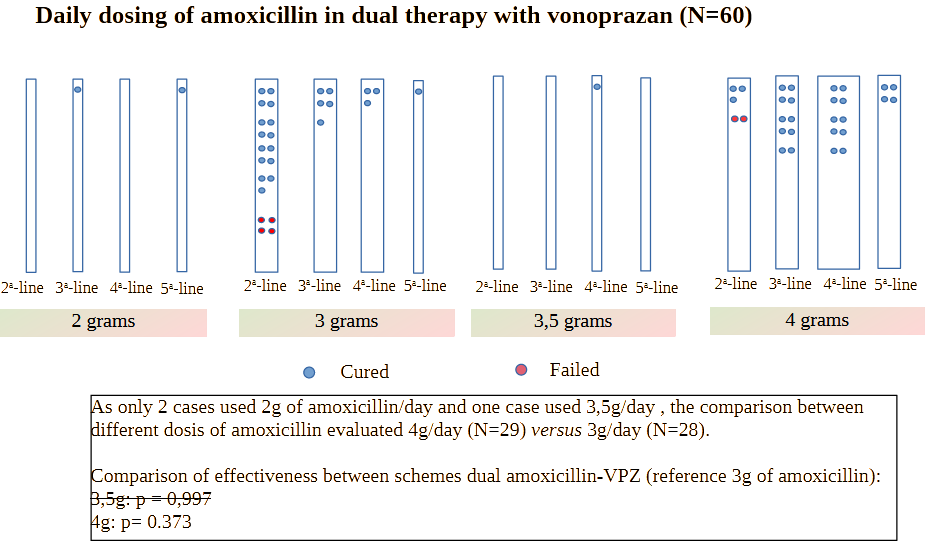


1. As only 2 cases used amoxicillin BID, the comparison between different times-daily evaluated QID (four times a day; N=30) against TID (three times a day; N=28)

Comparison of effectiveness between times-daily doses of amoxicillin in dual therapy with VPZ (reference TID of amoxicillin):

QID (four times a day): p = 0.352


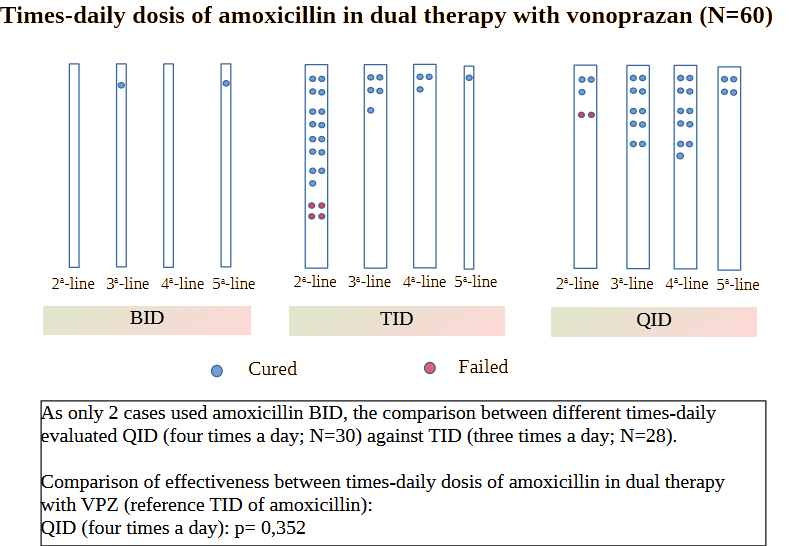

Supplement: Supplementary file 1 — Figures S1–S2: hel70077‐sup‐0001‐AppendixS1.docx. [file HEL-30-e70077-s002.docx]
