# Supplementary material for: Empirical Rescue Eradication Therapy for Helicobacter pylori Infection in Second and Subsequent Treatment Lines: Experience From 500 Cases of the Brazilian Registry on H. pylori Management (Hp‐BrazilReg)
Source: Helicobacter. 2025 Oct 14;30(5):e70077. doi: 10.1111/hel.70077 (PMC12521799; doi:10.1111/hel.70077)
Supplement: Supplementary file 2 — Appendix S1: hel70077‐sup‐0002‐AppendixS2.docx. [file HEL-30-e70077-s001.docx]

**Table S1. All empirical schemes reported in second-line treatment for *Helicobacter pylori* infection**

|  | Brazil | South-Eastern | Southern | North-Eastern | Northern | Central-Western |
| --- | --- | --- | --- | --- | --- | --- |
| A + VNZ | 24 (6.2%) | 6 (3.7%) | 13 (14.3%) | 4 (6.8%) | - | 1 (4%) |
| A + L + PPI | 212 (55%) | 85 (52.5%) | 18 (19.8%) | 38 (64.4%) | 49 (100%) | 22 (88%) |
| A + L + VNZ | 18 (4.7%) | 5 (3.1%) | 7 (7.7%) | 6 (10.2%) | - | - |
| A + M + PPI | 1 (0.3%) | 1 (.06%) | - | - | - | - |
| A + M + VNZ | 1 (0.3%) | 1 (0.6%) | - | - | - | - |
| Bi + A + L + PPI | 31 (8%) | 23 (14.2%) | 8 (8.8%) | - | - | - |
| Bi + A + L + VNZ | 5 (1.3%) | - | 5 (5.5%) | - | - | - |
| Bi + A + M + PPI | 1 (0.3%) | 1 (0.6%) | - | - | - | - |
| Bi + C + A + PPI | 2 (0.5%) | 2 (1.2%) | - | - | - | - |
| Bi + M + D + PPI | 5 (1.3%) | 5 (3.1%) |  |  |  | - |
| Bi + M + D + VNZ | 2 (0.5% | 2 (1.2%) | - | - | - | - |
| Bi + M + L + D + | 1 (0.3%) | - | 1 (1.1%) | - | - | - |
| PPI |  |  |  |  |  |  |
| Bi + M + Tc + PPI | 17 (4.4%) | 6 (3.7%) | 10 (11%) | - | - | 1 (4%) |
| Bi + M + Tc + VNZ | 13 (3.4%) | 1 (0.6%) | 12 (13.2%) | - | - | - |
| C + A + PPI | 37 (9.6%) | 23 (14.2%) | 12 (13.2%) | 1 (1.7%) | - | 1 (4%) |
| C + A + M + PPI | 1 (0.3%) | - | 1 (1.1%) | - | - | - |
| C + A + VNZ | 7 (1.8%) | - | 3 (3.3%) | 4 (6.8%) | - | - |
| C + L + PPI | 4 (1%) | - | - | 4 (6.8%) | - | - |
| C + L + VNZ | 1 (0.3%) | - | - | 1 (1.7%) | - | - |
| C + M + PPI | 1 (0.3%) | - | 1 (1.1%) | - | - | - |
| Single-capsule^†^ +  PPI | 1 (0.3%) | 1 (0.6%) | - | - | - | - |
| M + D + VNZ | 1 (0.3%) | - | - | 1 (1.7%) |  |  |

## Overall 386 (100%) 162 91 59 49 25



^†^ Three-in-one single-capsule containing bismuth, tetracycline and metronidazole.

A, amoxicillin; Bi, bismuth; C, clarithromycin; D, doxycicline; L, levofloxacin; M, metronidazole; PPI, proton-pump inhibitor; Tc, tetracycline hydrochloride; VNZ, vonoprazan.

**Table S2. All empirical schemes reported in third-line treatment for *Helicobacter pylori***

# **infection**

|  | Brazil | South-Eastern | Southern | North Eastern | Northern | Central-Westernn |
| --- | --- | --- | --- | --- | --- | --- |
| A + L + PPI | 13 (10%) | 7 (12.5%) | 5 (22.7%) | 1 (4.3%) | - | - |
| A + L + VNZ | 1 (0.8%) | - | 1 (4.5%) | - | - | - |
| A + M + PPI | 1 (0.8%) | 1 (1.8%) | - | - | - | - |
| A + M + VNZ | 2 (1.5%) | 2 (3.6%) | - | - | - | - |
| A + VNZ | 16 (12.3%) | 2 (3.6%) | 1 (4.5%) | 12 (52.2%) | - | 1 (11.1%) |
| Bi + A + D + PPI | 20 (15.4%) | - | - | - | 20 (100%) | - |
| Bi + A + M + PPI | 1 (0.8%) | 1 (1.8%) | - | - | - | - |
| Bi + A + M + VNZ | 1 (0.8%) | 1 (1.8%) | - | - | - | - |
| Bi + A + R + VNZ | 3 (2.3%) | 3 (5.4%) | - | - | - | - |
| Bi + C + A + PPI | 1 (0.8%) | 1 (1.8%) | - | - | - | - |
| Bi +M + D + NR | 1 (0.8%) | 1 (1.8%) |  |  |  |  |
| Bi + D + Tn + PPI | 1 (0.8%) | - | - | 1 (4.3%) | - | - |
| Bi + M + D + PPI | 10 (7.7%) | 4 (7.1%) |  | 6 (26.1%) |  |  |
| Bi + M + D + VNZ | 3 (2.3%) | - | 3 (13.6%) | - | - | - |
| Bi + M + Tc + PPI | 31 (23.8%) | 19 (33.9%) | 3 (13.6%) | 1 (4.3%) | - | 8 (88.9%) |
| Bi + M + Tc + VNZ | 10 (7.7%) | 4 (7.1%) | 6 (27.3%) | - | - | - |
| Bi + Tc + VNZ | 1 (0.8%) | - | 1 (4.5%) | - | - | - |
| C + A + PPI | 7 (5.4%) | 6 (10.7%) | 1 (4.5%) | - | - | - |
| C + A + M + PPI | 1 (0.8%) | 1 (1.8%) | - | - | - | - |
| C + A + Tn+ PPI | 2 (1.5%) | 2 (3.6%) | - | - | - | - |
| C + A + VNZ | 1 (0.8%) | - | - | 1 (4.3%) | - | - |
| C + L + PPI | 1 (0.8%) | - | - | 1 (4.3%) | - | - |
| C + Tc + VNZ | 1 (0.8%) | - | 1 (4.5%) | - | - | - |
| Single-capsule^†^ + PPI | 1 (0.8%) | 1 (1.8%) | - | - | - | - |
| Overall | 130 | 56 | 22 | 23 | 20 | 9 |



^†^ Three-in-one single-capsule containing bismuth, tetracycline and metronidazole.

A, amoxicillin; Bi, bismuth; C, clarithromycin; D, doxycicline; L, levofloxacin; M, metronidazole; N, nitazoxanide; PPI, proton-pump inhibitor; R, rifabutin; Tc, tetracycline hydrochloride; Tn, tinidazole; VNZ, vonoprazan.

# **Table S3. All empirical schemes reported in fourth-line treatment for *Helicobacter pylori* infection**

|  | Brazil | South-Eastern | Southern | North Eastern | Northern | Central-Western |
| --- | --- | --- | --- | --- | --- | --- |
| A + PPI | 2 (4.8%) | - | - | 2 (33.3%) | - | - |
| A + L + PPI | 1 (2.4%) | 1 (6.7%) | - | - | - | - |
| A + L + VNZ | 5 (11.9%) | - | 5 (55.6%) | - | - | - |
| A + VNZ | 14 (33.3%) | 2 (13.3%) | - | 1 (16.7%) | 9 (90%) | 2 (100%) |
| Bi + A + R + PPI | 1 (2.4%) | 1 (6.7%) | - | - | - | - |
| Bi + A + R + VNZ | 6 (14.3%) | 6 (40%) | - | - | - | - |
| Bi + M + D + PPI | 2 (4.8%) | - | - | 2 (33.3%) |  |  |
| Bi + M + Tc + PPI | 2 (4.8%) | 1 (6.7%) | - | 1 (16.7%) |  |  |
| Bi + M + Tc + VNZ | 1 (2.4%) | - | 1 (11.1%) | - | - | - |
| C + A + PPI | 2 (4.8%) | 1 (6.7%) | - | - | 1 (10%) | - |
| C + A + Tn + PPI | 1 (2.4%) | 1 (6.7%) | - | - | - | - |
| C + A + VNZ | 2 (4.8%) | - | 2 (22.2%) | - | - | - |
| Single-capsule^†^ + | 1 (2.4%) | 1 (6.7%) | - | - | - | - |
| PPI  L + D + N + PPI | 1 (2.4%) | - | 1 (11.1%) | - | - | - |
| M + T + PPI | 1 (2.4%) | 1 (6.7%) | - | - | - | - |
| Overall | 42 (100%) | 15 | 9 | 6 | 10 | 2 |

^†^ Three-in-one single-capsule containing bismuth, tetracycline and metronidazole.

A, amoxicillin; Bi, bismuth; C, clarithromycin; D, doxycicline; L, levofloxacin; M, metronidazole; N, nitazoxanide; PPI, proton-pump inhibitor; R;, rifabutin; T, tetracycline hydrochloride; Tn, tinidazole; VNZ, vonoprazan.

# **Table S4. All empirical schemes reported in fifth-line or more treatment for**

***Helicobacter pylori* infection**

|  | Brazil | South-Eastern | Southern | North Eastern | Northern | Central-Western |
| --- | --- | --- | --- | --- | --- | --- |
| A + PPI | 1 (7.1%) | - | - | 1 (16.7%) | - | - |
| A + L + PPI | 1 (7.1%) | 1 (16.7%) | - | - | - | - |
| A + VNZ | 6 (42.9%) | 2 (33.3%) | - | 4 (66.7%) | - | - |
| Bi + M + Tc + PPI | 1 (7.1%) | 1 (16.7%) | - | - | - | - |
| Bi + M + D + VNZ | 1 (7.1%) | - | 1 (50%) | - | - | - |
| Bi + Tc + Tn + PPI | 1 (7.1%) | - | - | 1 (16.7%) | - | - |
| C + A + PPI | 1 (7.1%) | 1 (16.7%) | - | - | - | - |
| C + A + Tn + PPI | 1 (7.1%) | 1 (16.7%) | - | - | - | - |
| C + A + VNZ | 1 (7.1%) | - | 1 (50%) | - | - | - |
| Overall | 14 (100%) | 6 | 2 | 6 | - | - |

A, amoxicillin; Bi, bismuth; C, clarithromycin; D, doxycicline; L, levofloxacin; M, metronidazole; N, nitazoxanide; PPI, proton-pump inhibitor; Tc, tetracycline hydrochloride; Tn, tinidazole; VNZ, vonoprazan.

| **Table S5. Effectiveness by modified intention-to-treat of the most commonly prescribed therapies by second-line treatment** | | | | | | | | | | | | | | | | | | | |
| --- | --- | --- | --- | --- | --- | --- | --- | --- | --- | --- | --- | --- | --- | --- | --- | --- | --- | --- | --- |
|  | | **Brazil** | | | **South-Eastern** | | | **Southern** | | | **North Eastern** | | | **Northern** | | | **Central-Western** | | |
| **Second line** | | Use, N | mITT, N | % (95% CI) | Use, N | mITT, N | % (95% CI) | Use, N | mITT, N | % (95% CI) | Use, N | mITT, N | % (95% CI) | Use. N | MITT, N | % (95%, CI) | Use. N | MITT, N | % (95%, CI) |
|  | | | | | | | | | | | | | | | | | | | |
| A + L + PPI | | 212 | 139 | 65.6 (59-72) | 85 | 60 | 70.6 (60-80) | 18 | 12 | 66.7 (41-87) | 38 | 23 | 60.5 (43-76) | 49 | 34 | 69.4 (55-82) | 22 | 10 | 45.5 (25-68) |
|  | 14 Days | 115 | 84 | 73 (64-81) | 77 | 54 | 70 (59-80) | 8 | 6 | 75 (35-97) | 3 | 3 | 100 (29-100) | 21 | 18 | 85.7 (64-97) | 6 | 3 | 50 (12-88) |
|  | 10 Days | 97 | 55 | 56.7 ((46-67) | 8 | 6 | 75 (35-97) | 10 | 6 | 60 (26-88) | 35 | 20 | 57.1 (39-74) | 28 | 16 | 57.1 (37-75) | 16 | 7 | 43.8 (20-70) |
|  | |  |  |  |  |  |  |  |  |  |  |  |  |  |  |  |  |  |  |
| C + A + PPI | | 37 | 28 | 75.7 (59-88) | 23 | 16 | 69.6 (47-87) | 12 | 10 | 83.3 (52-98) | 1 | 1 | 100 (2.5-100) | - | - | - | 1 | 1 | 100 (2.5-100) |
| Bi + A + L + PPI | | 31 | 31 | 100 (88-100) | 23 | 23 | 100 (85-100) | 8 | 8 | 100 (63-100) | - | - | - | - | - | - | - | - | - |
| A + VPZ | | 24 | 18 | 75 (53-90) | 6 | 6 | 100 (54-100) | 13 | 9 | 69 (38-90) | 4 | 2 | 50 (7-93) | - | - | - | 1 | 1 | 100 (2.5-100) |
| A + L + VPZ | | 18 | 16 | 88.9 (65-98) | 5 | 5 | 100 (48-100) | 7 | 5 | 71.4 (30-96) | 6 | 6 | 100 (54-100) | - | - | - | - | - | - |
| Bi + M + Tc + PPI | | 17 | 13 | 76 (50-93) | 6 | 3 | 50 (12-88) | 10 | 9 | 90 (56-99) |  |  |  |  |  |  | 1 | 1 | 100 (2.5-100) |
| Bi + M + Tc + VPZ | | 13 | 12 | 92.3 (64-100) | 1 | 1 | 100 (2.5-100) | 12 | 11 | 91.7 (61-99) | - | - | - | - | - | - | - | - | - |
| C + A + VPZ | | 7 | 6 | 85.7 (42-99) | - | - | - | 3 | 3 | 100 (29-100) | 4 | 3 | 75 (19-99) | - | - | - | - | - | - |
| Bi + A + L + VPZ | | 5 | 4 | 80 (28-99) | - | - | - | 5 | 4 | 80 (28-99) | - | - | - | - | - | - | - | - | - |
| Bi + M + D + PPI | | 5 | 5 | 100 (48-100) | 5 | 5 | 100 (48-100) | - | - | - | - | - | - | - | - | - | - | - | - |
| C + L + PPI | | 4 | 2 | 50 (7-93) | - | - | - | - | - | - | 4 | 2 | 50 (7-93) | - | - | - | - | - | - |
| Bi + C + A + PPI | | 2 | 2 | 100 (16-100) | 2 | 2 | 100 (16-100) | - | - | - | - | - | - | - | - | - | - | - | - |
| Bi + M + D + VPZ | | 2 | 2 | 100 (16-100) | 2 | 2 | 100 (16-100) | - | - | - | - | - | - | - | - | - | - | - | - |
| A + M + PPI | | 1 | 1 | 100 (2.5-100) | 1 | 1 | 100 (2.5-100) | - | - | - | - | - | - | - | - | - | - | - | - |
| A + M + VPZ | | 1 | 0 | 0 (-) | 1 | 0 | 0 (-) | - | - | - | - | - | - | - | - | - | - | - | - |
| Bi + A + M + PPI | | 1 | 1 | 100 (2.5-100) | 1 | 1 | 100 (2.5-100) | - | - | - | - | - | - | - | - | - | - | - | - |
| Bi + M + L + D + PPI | | 1 | 1 | 100 (2.5-100) | - | - | - | 1 | 1 | 100 (2.5-100) | - | - | - | - | - | - | - | - | - |
| C + A + M + PPI | | 1 | 1 | 100 (2.5-100) | - | - | - | 1 | 1 | 100 (2.5-100) | - | - | - | - | - | - | - | - | - |
| C + L + VPZ | | 1 | 1 | 100 (2.5-100) | - | - | - | - | - | - | 1 | 1 | 100 (2.5-100) | - | - | - | - | - | - |
| C + M + PPI | | 1 | 1 | 100 (2.5-100) |  |  |  | 1 | 1 | 100 (2.5-100) | - | - | - | - | - | - | - | - | - |
| Single-capsule^†^ + PPI | | 1 | 1 | 100 (2.5-100) | 1 | 1 | 100 (2.5-100) | - | - | - | - | - | - | - | - | - | - | - | - |
| M + D + VPZ | | 1 | 1 | 100 (2.5-100) |  |  |  | - | - | - | 1 | 1 | 100 (2.5-100) | - | - | - | - | - | - |
| Overall | | 386 | 286 | 73.6 (69-78) | 162 | 126 | 76.5 (69-82) | 91 | 74 | 81.3 (72-89) | 59 | 39 | 66.1 (53-78) | 49 | 34 | 69.4 (55-82) | 25 | 13 | 52 (31-72) |
| ^†^ Three-in-one single-capsule containing bismuth, tetracycline hydrochloride and metronidazole  A, amoxicillin; Bi, bismuth; C, clarithromycin; D, Doxycycline; L, levofloxacin; M, metronidazole; PPI, proton-pump inhibitor; R, rifabutin; Tc, tetracycline hydrochloride; Tn; tinidazole; VPZ, vonoprazan | | | | | | | | | | | | | | | | | | | |

| **Table S6. Effectiveness (by mITT) of the most commonly prescribed therapies by third-line treatment** | | | | | | | | | | | | | | | | | | | |
| --- | --- | --- | --- | --- | --- | --- | --- | --- | --- | --- | --- | --- | --- | --- | --- | --- | --- | --- | --- |
|  | | **Brazil** | | | **South-Eastern** | | | **Southern** | | | **North Eastern** | | | **Northern** | | | **Central-Western** | | |
| **Third-line** | | Use, N | mITT, N | % (95% CI) | Use, N | mITT, N | % (95% CI) | Use, N | mITT, N | % (95% CI) | Use, N | mITT, N | % (95% CI) | Use. N | MITT, N | % (95%, CI) | Use. N | MITT, N | % (95%, CI) |
| Bi + M + Tc + PPI | | 31 | 27 | 87 (70-96) | 19 | 17 | 89 (67-98) | 3 | 2 | 67 (9-99) | 1 | 1 | 100 (2.5-100) | - | - | - | 8 | 7 | 87 (47-99) |
| Bi + A + D + PPI | | 20 | 13 | 65 (41-85) | - | - | - | - | - | - | - | - | - | 20 | 13 | 65 (41-85) | - | - | - |
| A + VPZ | | 16 | 16 | 100 (80-100) | 2 | 2 | 100 (16-100) | 1 | 1 | 100 (2.5-100) | 12 | 12 | 100 (73-100) | - | - | - | 1 | 1 | 100 (2.5-100) |
| A + L + PPI | | 13 | 4 | 31 (9-61) | 7 | 3 | 43 (18-81) | 5 | 1 | 20 (0.5-71) | 1 | 0 | 0 -) | - | - | - | - | - | - |
|  | 14 Days | 10 | 3 | 30 (7-65) | 6 | 3 | 50 (12-88) | 4 | 0 | 0 (-) | - | - | - | - | - | - | - | - | - |
|  | 10 Days | 3 | 1 | 33 (8.4-90) | 1 | 0 | 0 (-) | 1 | 1 | (2.5-100) | 1 | 0 | 0 (-) | - | - | - | - | - | - |
| Bi + M + D + PPI | | 10 | 8 | 80 (44-97) | 4 | 4 | 100 (40-100) | - | - | - | 6 | 4 | 67 (22-95) | - | - | - | - | - | - |
| Bi + M + Tc + VPZ | | 10 | 9 | 90 (55-99) | 4 | 4 | 100 (39-100) | 6 | 5 | 83 (36-99) | - | - | - | - | - | - | - | - | - |
| C + A + PPI | | 7 | 5 | 71 (29-96) | 6 | 5 | 83 (36-99) | 1 | 0 | 0 (-) | - | - | - | - | - | - | - | - | - |
| Bi + M + D + VPZ | | 3 | 3 | 100 (29-100) | - | - | - | 3 | 3 | 100 (29-100) | - | - | - | - | - | - | - | - | - |
| Bi + A + R + VPZ | | 3 | 3 | 100 (29-100) | 3 | 3 | 100 (29-100) | - | - | - | - | - | - | - | - | - | - | - | - |
| C + A + Tn + PPI | | 2 | 2 | 100 (16-100) | 2 | 2 | 100 (16-100) | - | - | - | - | - | - | - | - | - | - | - | - |
| A + M + VPZ | | 2 | 1 | 50 (1.2-98) | 2 | 1 | 50 (1.2-98) | - | - | - | - | - | - | - | - | - | - | - | - |
| A + L + VPZ | | 1 | 0 | 0 (-) | - | - | - | 1 | 0 | 0 (-) |  |  |  | - | - | - | - | - | - |
| A + M + PPI | | 1 | 1 | 100 (2.5-100) | 1 | 1 | 100 (2.5-100) | - | - | - | - | - | - | - | - | - | - | - | - |
| Bi + A + M + PPI | | 1 | 0 | 0 (-) | 1 | 0 | 0 (-) | - | - | - | - | - | - | - | - | - | - | - | - |
| Bi + A + M + VPZ | | 1 | 1 | 100 (2.5-100) | 1 | 1 | 100 (2.5-100) | - | - | - | - | - | - | - | - | - | - | - | - |
| Bi + C + A + PPI | | 1 | 0 | 0 (-) | 1 | 0 | 0 (-) | - | - | - | - | - | - | - | - | - | - | - | - |
| Bi + D + Tn + PPI | | 1 | 0 | 0 (-) | - | - | - | - | - | - | 1 | 0 | 0 (-) | - | - | - | - | - | - |
| Bi + M + D + NR | | 1 | 1 | 100 (2.5-100) | 1 | 1 | 100 (2.5-100) | - | - | - | - | - | - | - | - | - | - | - | - |
| Bi + Tc + VPZ | | 1 | 0 | 0 (-) | - | - | - | 1 | 0 | 0 (-) | - | - | - | - | - | - | - | - | - |
| C + A + M + PPI | | 1 | 1 | 100 (2.5-100) | 1 | 1 | 100 (2.5-100) | - | - | - | - | - | - | - | - | - | - | - | - |
| C + A + VPZ | | 1 | 1 | 100 (2.5-100) | - | - | - | - | - | - | 1 | 1 | 100 (2.5-100) | - | - | - | - | - | - |
| C + L + PPI | | 1 | 0 | 0 (-) | - | - | - | - | - | - | 1 | 0 | 0 (-) | - | - | - | - | - | - |
| C + Tc + VPZ | | 1 | 1 | 100 (2.5-100) | - | - | - | 1 | 1 | 100 (2.5-100) | - | - | - | - | - | - | - | - | - |
| Single-capsule^†^ + PPI | | 1 | 1 | 100 (2.5-100) | 1 | 1 | 100 (2.5-100) | - | - | - | - | - | - | - | - | - | - | - | - |
|  | |  |  |  |  |  |  |  |  |  |  |  |  |  |  |  |  |  |  |
| Overall | | 130 | 98 | 75.4 (67-82) | 56 | 46 | 82 (69-91) | 22 | 13 | 59 (36-79) | 23 | 18 | 78 (56-92) | 20 | 13 | 65 (41-85) | 9 | 8 | 89 (51-99) |
| ^†^ Three-in-one single-capsule containing bismuth, tetracycline hydrochloride and metronidazole  A, amoxicillin; Bi, bismuth; C, clarithromycin; D, Doxycycline; L, levofloxacin; M, metronidazole; PPI, proton-pump inhibitor; R, rifabutin; Tc, tetracycline hydrochloride; Tn; tinidazole; VPZ, vonoprazan | | | | | | | | | | | | | | | | | | | |

| **Table S7 Effectiveness by intention-to-treat of the most commonly prescribed therapies by fourth-line treatment** | | | | | | | | | | | | | | | | | | |
| --- | --- | --- | --- | --- | --- | --- | --- | --- | --- | --- | --- | --- | --- | --- | --- | --- | --- | --- |
|  | **Brazil** | | | **South-Eastern** | | | **Southern** | | | **North Eastern** | | | **Northern** | | | **Central-Western** | | |
| **Fourth-line** | Use, N | mITT, N | % (95% CI) | Use, N | mITT, N | % (95% CI) | Use, N | mITT, N | % (95% CI) | Use, N | mITT, N | % (95% CI) | Use. N | MITT, N | % (95%, CI) | Use. N | MITT, N | % (95%, CI) |
| A + VPZ | 14 | 14 | 100 (77-100) | 2 | 2 | 100 (16-100) | - | - | - | 1 | 1 | 100 (2.5-100) | 9 | 9 | 100 (66-100) | 2 | 2 | 100 (16-100) |
| Bi + A + R + VPZ | 6 | 6 | 100 (54-100) | 6 | 6 | 100 (54-100) | - | - | - | - | - | - | - | - | - | - | - | - |
| A + L + VPZ | 5 | 3 | 60 (15-95) | - | - | - | 5 | 3 | 60 (15-95) | - | - | - | - | - | - | - | - | - |
| A + PPI | 2 | 0 | 0 (-) | - | - | - | - | - | - | 2 | 0 | 0 (-) | - | - | - | - | - | - |
| Bi + M + Tc + PPI | 2 | 2 | 100 (16-100) | 1 | 1 | 100 (2.5-100) | - | - | - | 1 | 1 | 100 (2.5-100) | - | - | - | - | - | - |
| Bi + M + D + PPI | 2 | 0 | 0 (-) | - | - | - | - | - | - | 2 | 0 | 0 (-) | - | - | - | - | - | - |
| C + A + PPI | 2 | 1 | 50 (12.5-99) | 1 | 0 | 0 (-) | - | - | - | - | - | - | 1 | 1 | 100 (2.5-100) | - | - | - |
| C + A + VPZ | 2 | 2 | 100 (16-100) | - | - | - | 2 | 2 | 100 (16-100) | - | - | - | - | - | - | - | - | - |
| A + L + PPI | 1 | 1 | 100 (2.5-100) | 1 | 1 | 100 (2.5-100) | - | - | - | - | - | - | - | - | - | - | - | - |
| Bi + A + R + PPI | 1 | 1 | 100 (2.5-100) | 1 | 1 | 100 (2.5-100) | - | - | - | - | - | - | - | - | - | - | - | - |
| Bi + M + Tc + VPZ | 1 | 1 | 100 (2.5-100) | - | - | - | 1 | 1 | 100 (2.5-100) | - | - | - | - | - | - | - | - | - |
| C + A + Tn + PPI | 1 | 1 | 100 (2.5-100) | 1 | 1 | 100 (2.5-100) | - | - | - | - | - | - | - | - | - | - | - | - |
| Single-capsule^†^ + PPI | 1 | 1 | 100 (2.5-100) | 1 | 1 | 100 (2.5-100) | - | - | - | - | - | - | - | - | - | - | - | - |
| L + D + N+ PPI | 1 | 1 | 100 (2.5-100) | - | - | - | 1 | 1 | 100 (2.5-100) | - | - | - | - | - | - | - | - | - |
| M + Tc + PPI | 1 | 1 | 100 (2.5-100) | 1 | 1 | 100 (2.5-100) | - | - | - | - | - | - | - | - | - | - | - | - |
|  |  |  |  |  |  |  |  |  |  |  |  |  |  |  |  |  |  |  |
| Overall | 42 | 35 | 83 (68-93) | 15 | 14 | 93 (68-99) | 9 | 7 | 78 (40-97) | 6 | 2 | 33 (4.3-77) | 10 | 10 | 100 (69-100) | 2 | 2 | 100 (16-100) |
| ^†^ Three-in-one single-capsule containing bismuth, tetracycline hydrochloride and metronidazole  A, amoxicillin; Bi, bismuth; C, clarithromycin; D, Doxycycline; L, levofloxacin; M, metronidazole; PPI, proton-pump inhibitor; R, rifabutin; Tc, tetracycline hydrochloride; Tn; tinidazole; VPZ, vonoprazan | | | | | | | | | | | | | | | | | | |

| **Table S8. Effectiveness by intention-to-treat of the most commonly prescribed therapies by fifth or more treatment** | | | | | | | | | | | | | | | | | | |
| --- | --- | --- | --- | --- | --- | --- | --- | --- | --- | --- | --- | --- | --- | --- | --- | --- | --- | --- |
|  | **Brazil** | | | **South-Eastern** | | | **Southern** | | | **North Eastern** | | | **Northern** | | | **Central-Western** | | |
| **Fifth-line and more** | Use, N | mITT, N | % (95% CI) | Use, N | mITT, N | % (95% CI) | Use, N | mITT, N | % (95% CI) | Use, N | mITT, N | % (95% CI) | Use. N | MITT, N | % (95%, CI) | Use. N | MITT, N | % (95%, CI) |
| A + VPZ | 6 | 6 | 100 (54-100) | 2 | 2 | 100 (16-100) | - | - | - | 4 | 4 | 100 (40-100) | - | - | - | - | - | - |
| A + PPI | 1 | 0 | 0 (-) | - | - | - | - | - | - | 1 | 0 | 0 (-) | - | - | - | - | - | - |
| A + L + PPI | 1 | 1 | 100 (2.5-100) | 1 | 1 | 100 (2.5-100) | - | - | - | - | - | - | - | - | - | - | - | - |
| Bi + M + Tc + PPI | 1 | 1 | 100 (2.5-100) | 1 | 1 | 100 (2.5-100) | - | - | - | - | - | - | - | - | - | - | - | - |
| Bi + M + D + VPZ | 1 | 1 | 100 (2.5-100) | - | - | - | 1 | 1 | 100 (2.5-100) | - | - | - | - | - | - | - | - | - |
| Bi + Tc + Tn + PPI | 1 | 1 | 100 (2.5-100) | - | - | - | - | - | - | 1 | 1 | 100 (2.5-100) | - | - | - | - | - | - |
| C + A + PPI | 1 | 0 | 0 (-) | 1 | 0 | 0 (-) | - | - | - | - | - | - | - | - | - | - | - | - |
| C + A + Tn + PPI | 1 | 1 | 100 (2.5-100) | 1 | 1 | 100 (2.5-100) | - | - | - | - | - | - | - | - | - | - | - | - |
| C + A + VPZ | 1 | 1 | 100 (2.5-100) | - | - | - | 1 | 1 | 100 (2.5-100) | - | - | - | - | - | - | - | - | - |
|  |  |  |  |  |  |  |  |  |  |  |  |  |  |  |  |  |  |  |
| Overall | 14 | 12 | 85 (57-98) | 6 | 5 | 83 (36-99) | 2 | 2 | 100 (16-100) | 6 | 5 | 83 (36-99) | - | - | - | - | - | - |
| A, amoxicillin; Bi, bismuth; C, clarithromycin; D, Doxycycline; L, levofloxacin; M, metronidazole; PPI, proton-pump inhibitor; Tc, tetracycline hydrochloride; Tn; tinidazole; VPZ, vonoprazan | | | | | | | | | | | | | | | | | | |

## **Table S9. Compliance in most frequently prescribed schemes**

|  | n/N | Compliance ≥ 90%  % (95% CI) |  |
| --- | --- | --- | --- |
| A + L + PPI | 225/227 | 99 (97-99) |  |
| A + VPZ | 60/60 | 100 (94-100) |  |
| Bi + M + Tc + PPI | 49/51 | 96 (86-99) |  |
| C + A + PPI | 47/47 | 100 (92.5-100) |  |
| Bi + A + L + PPI | 31/31 | 100 (88-100) |  |
| Bi + M + Tc + VPZ | 24/24 | 100 (86-100) |  |
| A + L + VPZ | 24/24 | 100 (86-100) |  |
| Bi + A + D + PPI | 20/20 | 100 (83-100) |  |
| Bi + M + D + PPI | 17/17 | 100 (80-100) |  |
| C + A + VPZ | 11/11 | 100 (71-100) |  |
| Bi + A + R + VPZ | 8/9 | 89 (52-99) |  |
| Bi + M + D + VPZ | 6/6 | 100 (54-100) |  |
| C + L + PPI | 5/5 | 100 (48-100) |  |
| Bi + A + L + VPZ | 5/5 | 100 (48-100) |  |
| C + A + Tn + PPI | 4/4 | 100 (40-100) |  |
| Single capsule^†^ + PPI | 3/3 | 100 (29-100) |  |
| Bi + C + A + PPI | 3/3 | 100 (29-100) |  |
| A + PPI | 3/3 | 100 (29-100) |  |
| A + M + VPZ | 3/3 | 100 (29-100) |  |
| **Overall** | 566/572 | 99 (97-99) |  |

^†^ Three-in-one single-capsule containing bismuth, tetracycline hydrochloride and metronidazole No adverse event in other schemes.

A, amoxicillin; Bi, bismuth; C, clarithromycin; D, doxycycline; L, levofloxacin; M, metronidazole;

PPI, proton-pump inhibitor; R, rifabutin; Tc, tetracycline hydrochloride; Tn; tinidazole; VPZ, vonoprazan

### **Table S10. Types of adverse events reported during retreatment therapy**

| **AEs** | **N (%)** | **Mean duration (days)** |
| --- | --- | --- |
| Any AE | 134 (23.4%) | 7 |
| Serious AEs | 0 | 0 |
| AEs leading discontinuation of medication | 0 | 0 |
| Nausea | 81 (14%) | 6 |
| Metallic taste | 48 (8.4%) | 6 |
| Diarrhea | 28 (4.9%) | 4 |
| Dyspepsia | 16 (2.8%) | 8 |
| Abdominal pain | 15 (2.6%) | 8 |
| Vomiting | 15 (2.6%) | 7 |
| Anorexia | 11 (1.9%) | 8 |
| Asthenia | 8 (1.4%) | 5 |
| Heartburn | 5 (0.9%) | 10 |

**Figure S1. Residual analysis in the logistical regression model to evaluate the effectiveness of *Helicobacter pylori* treatment**


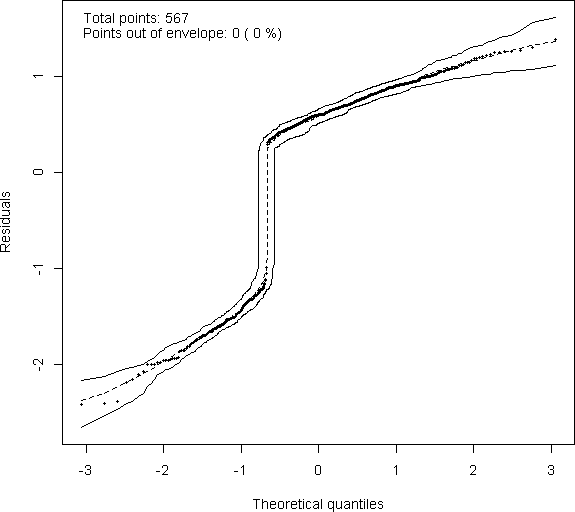


Figure S1. Residual analysis in the logistic regression model to evaluate the effectiveness of Helicobacter pylori treatment show the model is adequate

**Figure S2– Daily dosing (a) and times-daily doses of amoxicillin (b) in dual therapy with vonoprazan (N=60)**

1. As only 2 cases used 2g of amoxicillin/day and one case used 3,5g/day, the comparison between different doses of amoxicillin evaluated 4g/day (N=29) versus 3g/day (N=28).

Comparison of effectiveness between schemes dual amoxicillin-VPZ (reference 3g of amoxicillin):

4g: p= 0.373

**
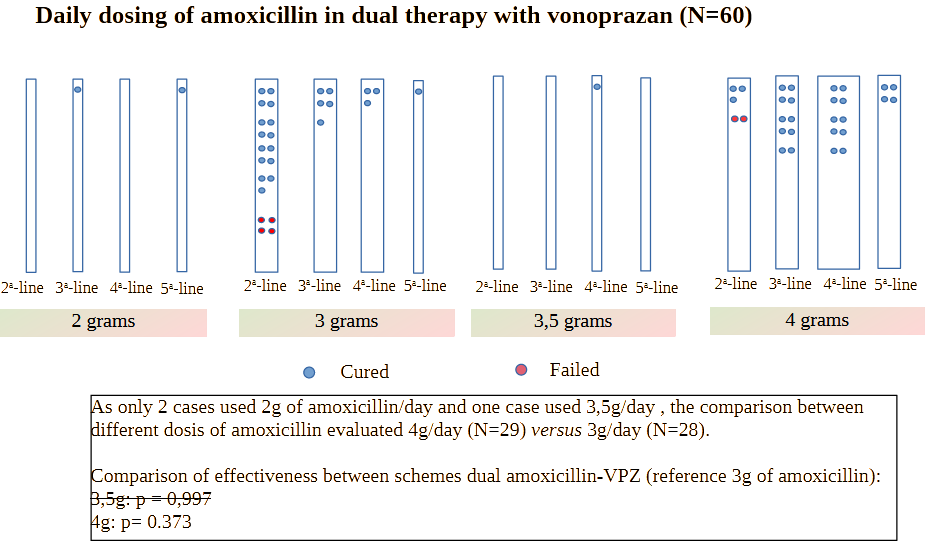
**

1. As only 2 cases used amoxicillin BID, the comparison between different times-daily evaluated QID (four times a day; N=30) against TID (three times a day; N=28)

Comparison of effectiveness between times-daily doses of amoxicillin in dual therapy with VPZ (reference TID of amoxicillin):

QID (four times a day): p = 0.352


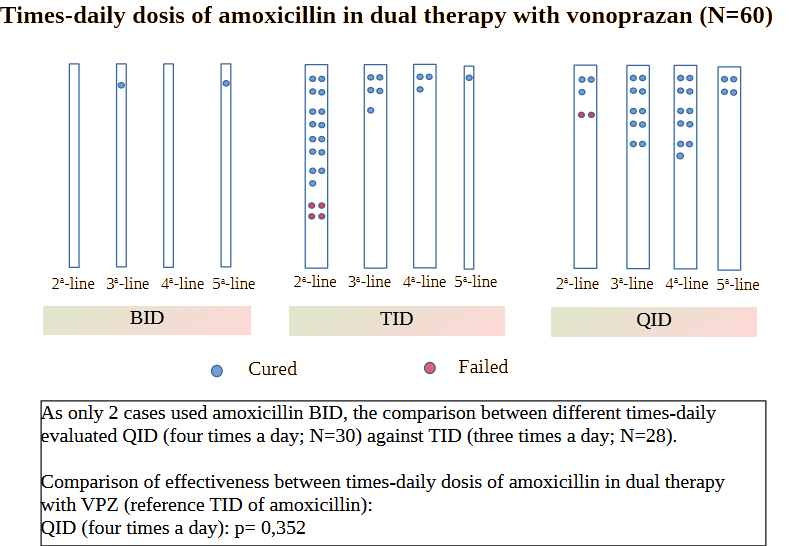


**Supplementary File S1.**

Collaborators: The Hp- BrazilReg Investigators:

S. N. Silveira^1^, J. Rezende-Filho^2^, C. F. Krumel^3^, A. C. G. G. Nespolo^4^, L. R. P. Oliveira^5^, A. C. Nascimento^6^, A. L. Hirt^7^, J. Farha^8^, L. F. Guidi^9^, A. S. Pinto^10^, A. J. B. M. Ramos^11^, C. J. L. Sá^12^, J. L. R. Farias^13^

^1^Private practice, Rio de Janeiro, Brazil; ^2^Universidade Federal de Goias, Belo Horizonte, Brazil; ^3^Universidade de Santa Cruz do Sul, Santa Cruz do Sul, Brazil; ^4^Centro Medico Cavalcante, Guarujá, Brazil; ^5^Centro Médico Monte Sinai, Juiz de Fora, Brazil; ^6^Clínica Santa Lourdes, Manaus, Brazil; ^7^Gastrocare, Curitiba, Brazil; ^8^Jorge Farha Gastroclínica, Rio de Janeiro, Brazil; ^9^Private practice, Nova Friburgo, Brazil; ^10^Hospital Santa Julia, Manaus, Brazil; ^11^Clínica Médica Dr Almiro, Chapecó, Brazil; ^12^Private practice, Palmeira dos Índios, Brazil; ^13^A. C. Camargo Cancer Center. São Paulo, Brazil.

All worked to collect data.

**Supplementary File 2 . Evaluation of data quality**

In order to maintain homogeneity of the collected data, as well as to maximize their use in statistical analyses, the Scientific Committee decided to make a selection of the most relevant variables of the e-CRF that would need to be completed in full.

These variables are requested to be filled in correctly in order to increase the number of high-quality records for analyses. This monitoring of data quality is performed firstly through a strategy in REDCap by means of an advanced “real-time” report which will detect all records with missing information on the following variables:

1. Line of treatment

2. Prescribed regimen

3. Duration of treatment

4. Prescribed drugs

5. Adherence to treatment

6. Result of post-treatment diagnostic tests (positive or negative)

7. Confirmation of eradication (success, failure or lost to follow-up) by the prescribing physician.

The technical project manager contacted the investigators for appropriate data correction on a quarterly basis.

An additional review of data were performed after each data extraction, reviewing at least 10% of the included patients. This second-level data review process evaluates whether the study selection criteria were met, whether information was correctly registered and ultimately to ensure the study was conducted according to the highest scientific and ethical standards. Additionally, prior to statistical analysis, data were reviewed for inconsistencies mainly on the variables described above checking for further detail. Data discordances were resolved by the technical project manager as well by the co-authors S.R.Chaves and J. N. Gonçalves (the statistician) querying the investigators and through group emailing.
